# Supplementary material for: Hexosamine biosynthetic pathway and O-GlcNAc-processing enzymes regulate daily rhythms in protein O-GlcNAcylation
Source: Nat Commun. 2021 Jul 7;12:4173. doi: 10.1038/s41467-021-24301-7 (PMC8263742; doi:10.1038/s41467-021-24301-7)
Supplement: Supplementary file 1 — Supplementary Information [file 41467_2021_24301_MOESM1_ESM.pdf]

Hexosamine biosynthetic pathway and O-GlcNAc-processing enzymes integrate circadian and metabolic signals to regulate daily rhythms in protein O-GlcNAcylation

Liu, X. *et al.*

Supplementary information: 5 Figures; 2 Tables; 3 Datasets, Source data

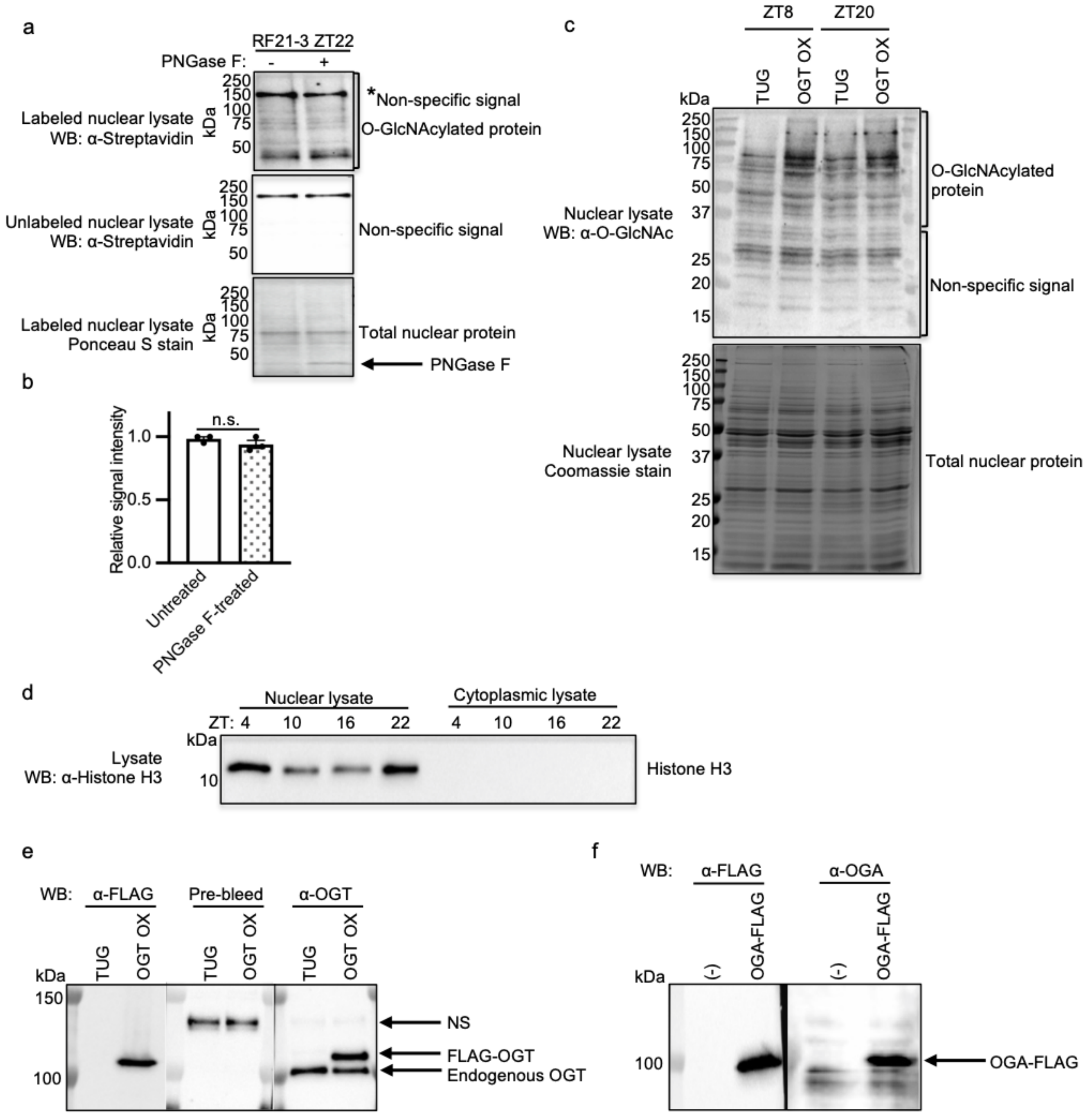

Supplementary Fig. 1. Validation of biochemical methods and antibodies. a Western blot detecting O-GlcNAcylated proteins in nuclear lysates treated with or without PNGase F. Protein O-GlcNAcylation was detected using chemoenzymatic labeling (top 2 panels) in combination with immunoblotting with α-streptavidin. Unlabeled samples (second panel) were processed in parallel to labeled samples (top panel) to identify non-specific signal for background deduction. Total

nuclear proteins stained by Ponceau S (bottom) were used for normalization. The asterisk (top panel) denotes non-specific signal. The arrow (third panel) indicates PNGase F. Each biological replicate was performed independently with similar results (n=3). **b** Quantification of protein O-GlcNAcylation detected using enzymatic labeling (n=3; p=0.27; two-tailed Student's t-test)**.** Data are normalized (untreated sample=1) and presented as mean ± SEM. **c** Validation of α-O-GlcNAc antibody to detect O-GlcNAcylated nuclear proteins in fly tissues. Flies overexpressing OGT in *timeless*-expressing clock cells (*w; tim(UAS)-gal4; UAS-FLAG-ogt*)[1], denoted as OGT OX, and parental control flies (*w; tim(UAS)-gal4,* denoted as *TUG*) were entrained in 12hrs light/ 12 hrs dark cycle at 25°C for 2 days and collected at ZT8 and 20 on LD3. Nuclear proteins were extracted from fly bodies and immunoblotting by α-O-GlcNAc was performed. Total nuclear proteins were stained by Coomassie blue (bottom panel) to indicate equal loading. Each biological replicate was performed independently with similar results (n=2). **d** Validation of nuclear-cytoplasmic protein fractionation in wild type flies (w^{1118}). Flies were entrained in 12hrs light/ 12 hrs dark cycle at 25°C for 2 days and collected at 4 time-points on LD3. Histone H3 was detected by immunoblotting to verify the purity of nuclear and cytoplasmic protein fractions. Each biological replicate was performed independently with similar results (n=2). **e, f** Validation of α-OGT and α-OGA antibodies to detect *Drosophila* OGT and OGA proteins. **e** Protein extracts from *TUG* and OGT OX flies were detected using α-FLAG to indicate the size of OGT-FLAG (left blot). Pre-bleed was used to indicate any nonspecific signals (middle blot). α-OGT antibody was able to detect both FLAG-tagged OGT and endogenous OGT (right blot). **f** *Drosophila* S2 cells expressing the empty vector pMT-FLAG (denoted as (-)) or pMT-*oga*-FLAG (denoted as OGA-FLAG) were used to validate α-OGA antibody. α-FLAG was used to indicate the size of OGA-FLAG (left blot). α-OGA antibody detected OGA in cells transfected with pMT-*oga*-FLAG but not in (-) sample (right blot). Each biological replicate was performed independently with similar results (n=2). Related to Figures 1, 3, 5 and 6.

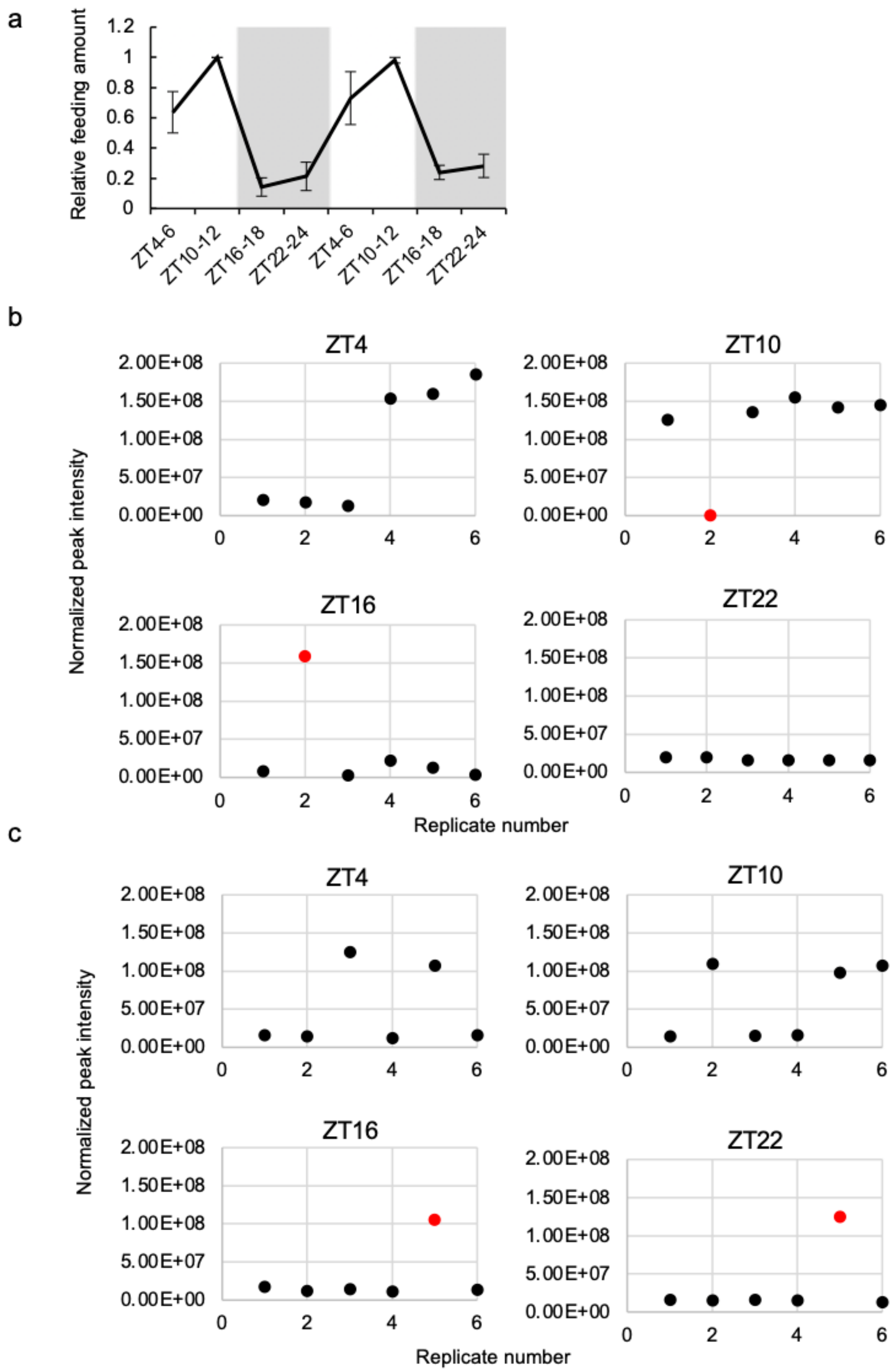

Supplementary Fig. 2. Analysis of fly feeding rhythms and circadian metabolomics. a Line graph showing the feeding-fasting cycles of WT (*w*^{1118}) female flies over 2 day-night cycles (n=3, 10 flies per biological replicate; p=6.96E-07, RAIN). Data were normalized (peak=1) and presented as mean ± SEM. **b-c** Determination of outlier replicates in GC-MS metabolomics to be

excluded from analysis. The quantity of the total metabolite peak intensity in each sample was graphed to identify outlier replicates in (**b**) body and (**c**) head samples. The red dots indicate outlier replicates. Related to Figure 2.

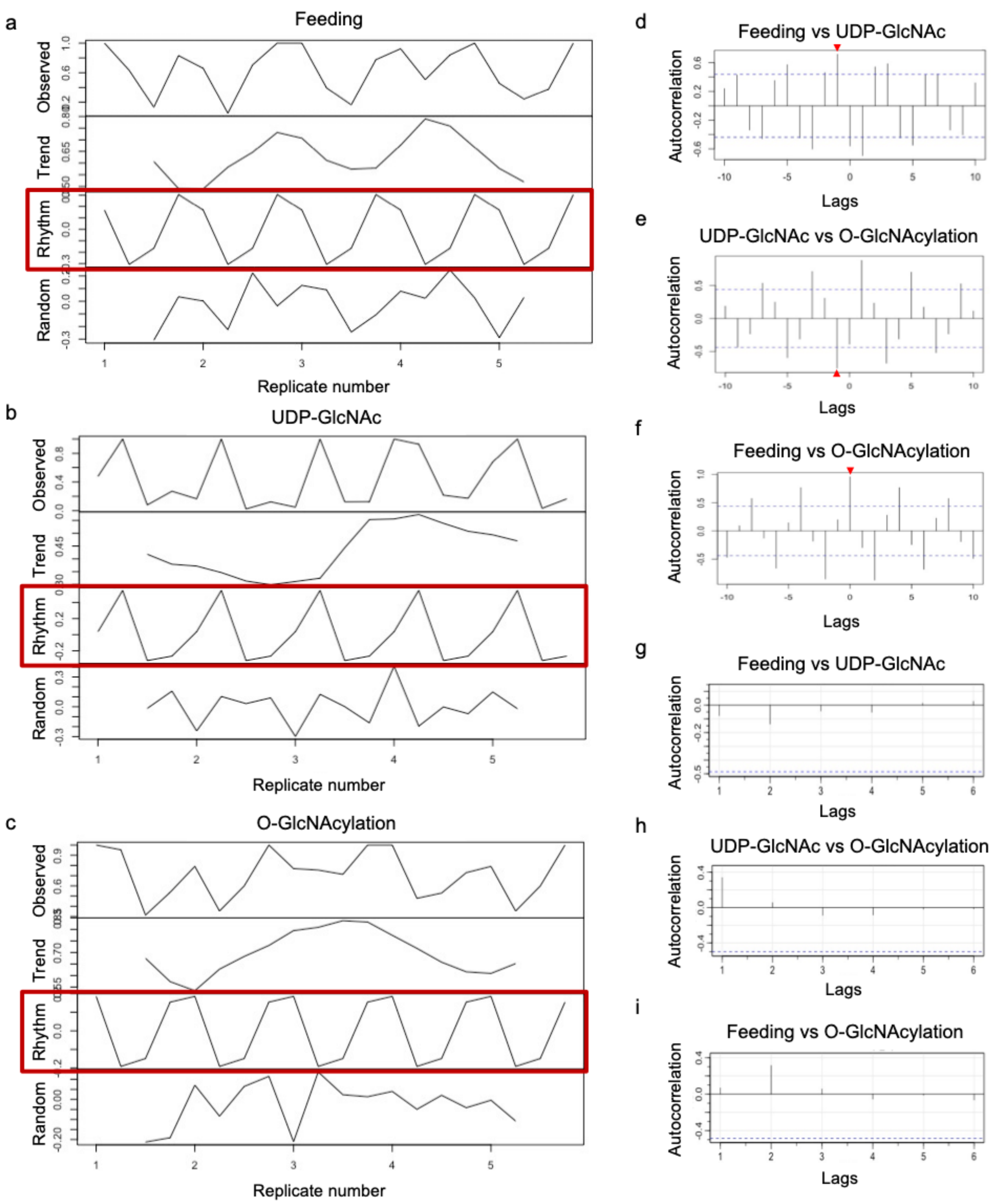

Supplementary Fig. 3. Cross correlation between the rhythms of feeding activity, UDP-GlcNAc, and nuclear protein O-GlcNAcylation. a-c Decomposition of observed raw data. Only rhythmic patterns of (**a**) feeding activity, (**b**) UDP-GlcNAc, and (**c**) O-GlcNAcylation were used for cross correlation analysis (boxed in red). **d-f** Calculation of cross correlation between rhythms.

(**d**) UDP-GlcNAc rhythm is significantly correlated to feeding rhythm by lag -1 (i.e. The current UDP-GlcNAc level can be predicted from feeding activity that is around 6 hours earlier). (**e**) O-GlcNAcylation rhythm is significantly correlated to UDP-GlcNAc rhythm also by lag -1, while (**f**) O-GlcNAcylation rhythm is significantly correlated to feeding rhythm by lag 0. Dash lines indicate the cutoff of significance. Red triangle indicates the lag where two rhythms are significantly correlated with each other. **g-i** Correlation of residuals from the predicted models (Fig. 2f-h). All the residuals are not significantly correlated with each other, indicating our models successfully predicted all the possible correlation between rhythms. Dashed lines indicate the cutoff of correlation significance. Related to Figure 2.

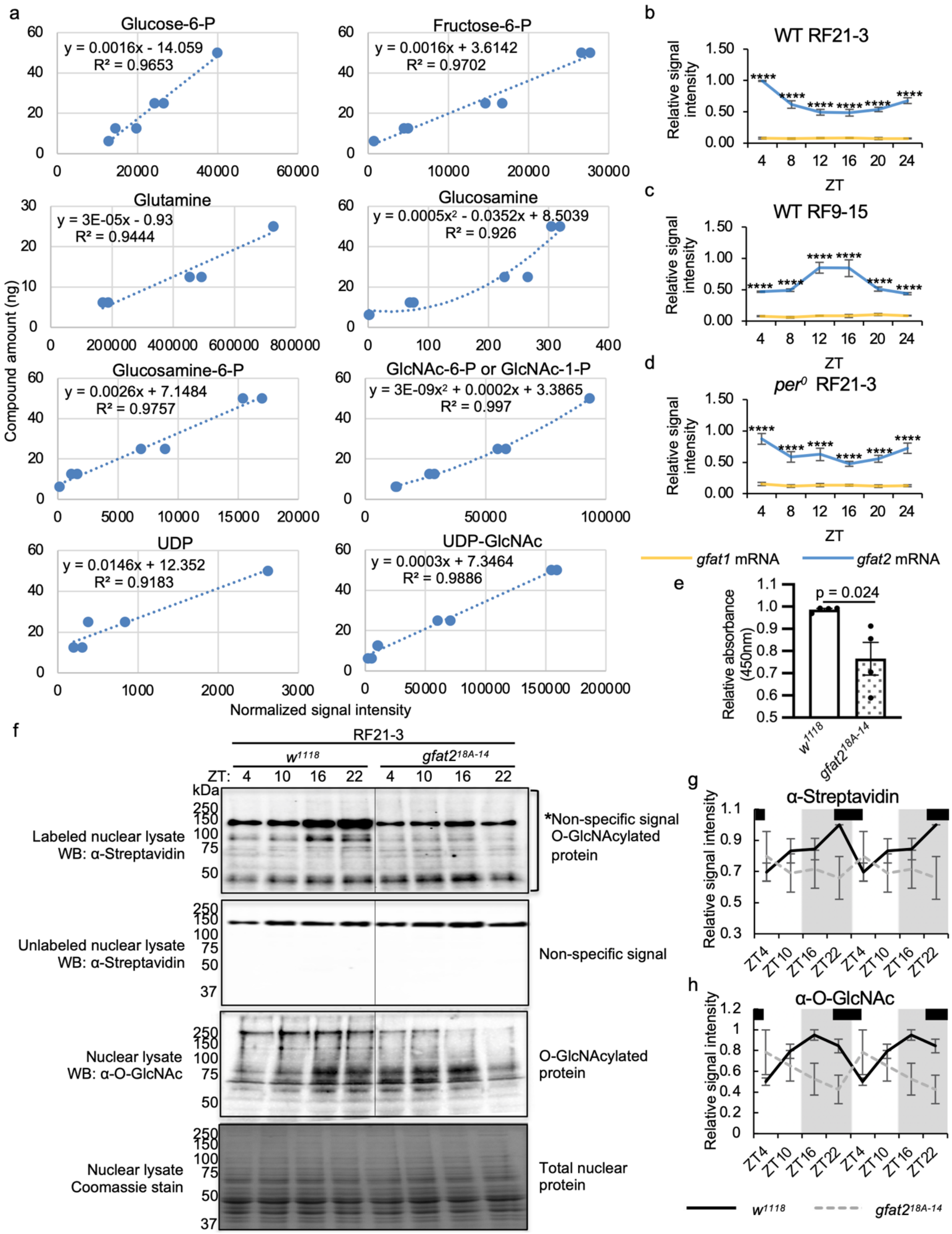

a
Glucose-6-P
y = 0.0016x - 14.059
R² = 0.9653
Fructose-6-P
y = 0.0016x + 3.6142
R² = 0.9702
Glutamine
y = 3E-05x - 0.93
R² = 0.9444
Glucosamine
y = 0.0005x² - 0.0352x + 8.5039
R² = 0.926
Glucosamine-6-P
y = 0.0026x + 7.1484
R² = 0.9757
GlcNAc-6-P or GlcNAc-1-P
y = 3E-09x² + 0.0002x + 3.3865
R² = 0.997
UDP
y = 0.0146x + 12.352
R² = 0.9183
UDP-GlcNAc
y = 0.0003x + 7.3464
R² = 0.9886
Compound amount (ng)
Normalized signal intensity
b
WT RF21-3
c
WT RF9-15
d
per0 RF21-3
Relative signal intensity
ZT
gfat1 mRNA
gfat2 mRNA
e
Relative absorbance (450nm)
p = 0.024
w1118
gfat218A-14
f
RF21-3
w1118
gfat218A-14
ZT: 4 10 16 22 4 10 16 22
kDa
Labeled nuclear lysate WB: α-Streptavidin
*Non-specific signal
O-GlcNAcylated protein
Unlabeled nuclear lysate WB: α-Streptavidin
Non-specific signal
Nuclear lysate WB: α-O-GlcNAc
O-GlcNAcylated protein
Nuclear lysate Coomassie stain
Total nuclear protein
g
α-Streptavidin
Relative signal intensity
h
α-O-GlcNAc
Relative signal intensity
ZT4 ZT10 ZT16 ZT22
w1118
gfat218A-14

Supplementary Fig. 4. Analysis of HBP metabolites and *gfat* mRNA expression. a Standard curves of HBP metabolites generated for targeted metabolomics analysis. The peak intensity of HBP metabolites in HILIC-MS is proportional to their concentration. The formula for each of the trend curves is indicated on the top left corner of each panel. **b-d** Expression level of *gfat2* is 5- to 10-fold higher than that of *gfat1* in fly bodies. Line graphs to compare *gfat1* and *gfat2* mRNA levels in (**b-c**) WT (w^{1118}) and (**d**) per^0 flies (n=3). Asterisks denote significant differences between *gfat1* and *gfat2* mRNA levels as determined by two-way ANOVA with *post-hoc* Tukey's HSD tests. ****p<0.0001. **e** Bar graph representing overall GFAT enzymatic activity in WT (w^{1118}) and *gfat2* heterozygous mutant ($gfat2^{18A\text{-}14}$/+) flies (n=4; p = 0.024; two-tailed Student's t-test). WT (w^{1118}) and $gfat2^{18A\text{-}14}$/+ flies were fed at natural feeding time (ZT21-3) and collected at ZT4 for GFAT activity assay. **f** Western blots showing daily rhythms in nuclear protein O-GlcNAcylation in WT (w^{1118}) and $gfat2^{18A\text{-}14}$/+ flies fed at natural feeding time. Protein O-GlcNAcylation was detected using two methods: (top 2 panels) chemoenzymatic labeling in combination with immunoblotting with α-streptavidin and (third panel) immunoblotting with α-O-GlcNAc. Unlabeled samples (second panel) were processed in parallel to labeled samples (top panel) to identify non-specific signal. Total nuclear proteins stained by Coomassie blue (bottom) were used for normalization. The asterisk (top panel) denotes non-specific signal. Each biological replicate was performed independently with similar results (n=4). **g-h** Quantification of protein O-GlcNAcylation detected using enzymatic labeling (n=4; WT: p=0.0023, $gfat2^{18A\text{-}14}$/+: p=0.83, RAIN), and using α-O-GlcNAc (n=4; WT: p=0.00023, $gfat2^{18A\text{-}14}$/+: p=0.24, RAIN). Data are normalized (peak=1) and presented as mean ± SEM. Data are double plotted. Gray shading indicates the dark period in LD cycle. Black bars on the top of graphs denote the restricted feeding periods for flies, ZT21 to ZT3. Related to Figure 4.

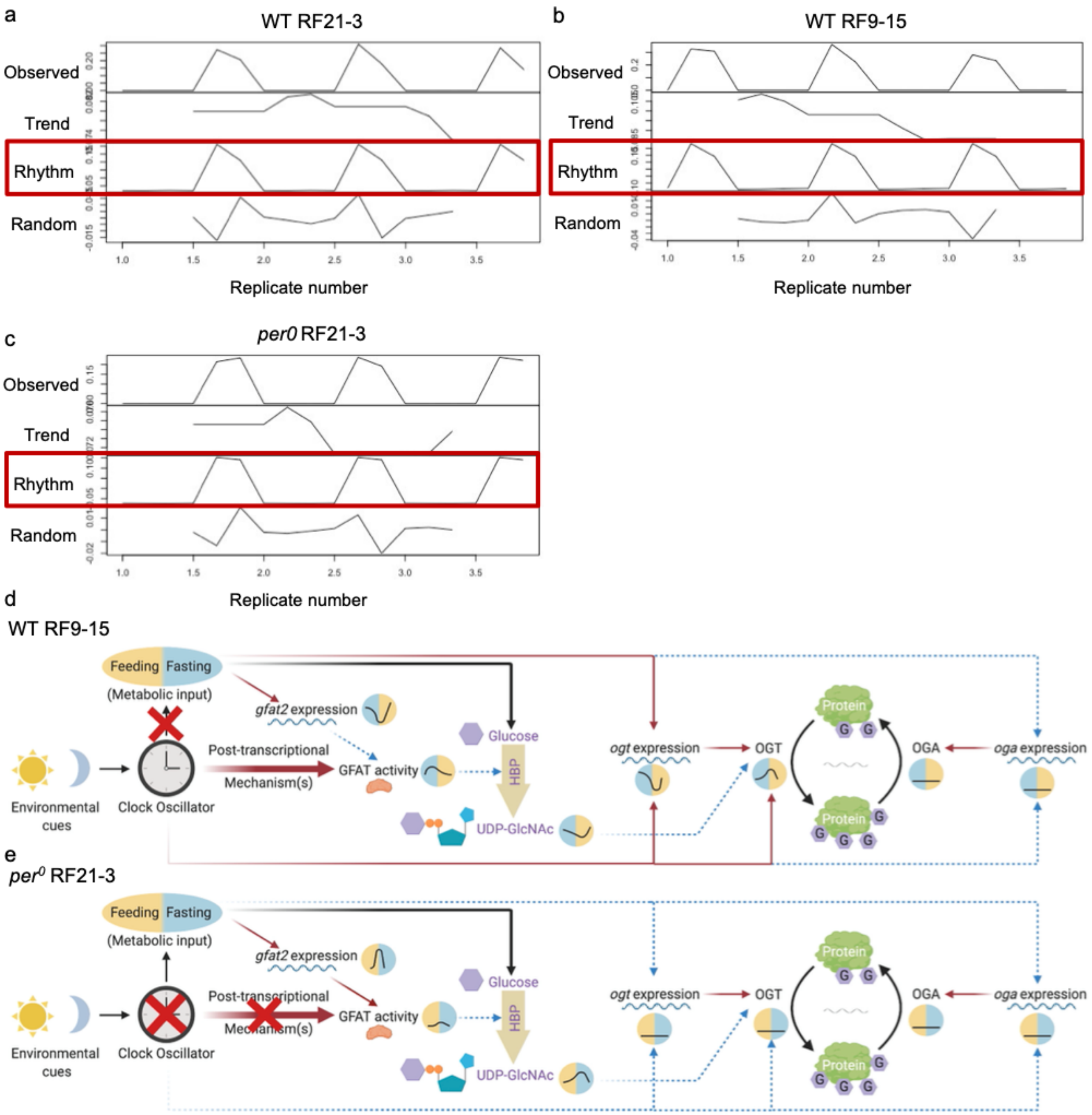

Supplementary Fig. 5. Decomposition of fly experimental data to predict the regulation of protein O-GlcNAcylation rhythm in flies. Observed feeding data of (**a**) WT RF21-3, (**b**) WT RF9-15, and (**c**) *per⁰* RF21-3 groups were decomposed in R. Only rhythmic patterns of the feeding activity, denoted by red boxes, were used as input for the mathematical model. **d** In WT RF9-15 flies, the clock-controlled post-transcriptional mechanism(s) still stimulates GFAT activity around

their natural feeding time. However, the food-induced *gfat2* expression occurs later, which decreases its contribution to GFAT activity. Consequently, there is less nutrient flow through HBP and reduced production of UDP-GlcNAc. Since OGT and OGA protein levels are regulated by the molecular clock and nutrient input, OGT and OGA protein rhythms are dampen when flies feed at unnatural feeding time. Together, the reduced UDP-GlcNAc level and dampened OGT protein rhythm result in the dampened protein O-GlcNAcylation rhythm. **f** In *per*^{0} RF21-3 flies, clock-controlled post-transcriptional mechanism(s) is(are) absent. Although nutrient influx can still increase the expression of *gfat2* in a rhythmic manner, GFAT activity cannot reach a high level due to the lack of modulation by post-transcriptional mechanism(s). Consequently, the low GFAT activity after fly feeding results in lower UDP-GlcNAc production. Since an intact molecular clock is necessary for OGT and OGA proteins to display robust daily oscillation, *per*^{0} RF21-3 flies exhibit arrhythmic OGT and OGA protein. As a result, O-GlcNAcylation rhythm is dampened. Yellow background indicates feeding period, while the blue background indicates fasting period. Red arrows denote activation, and the thicker red arrows denote a stronger effect. Blue dashed arrows denote reduced effect of the indicated factors. Related to Figure 7.

Supplementary Table 1. Circadian regulation of hexosamine biosynthetic pathway and O-GlcNAc cycling in fly head or mouse liver tissues based on published data.

Enzymes	Fly head		Mouse liver			Reference
	Transcripts	Protein	Transcripts	Protein	Phospho-peptide	
GFAT/GFPT	Cycling (18.4)	Non-cycling	Cycling (2.3)	Non-cycling	Cycling (4.7)	2-6
GNPNAT	Cycling (12.6)	Cycling (5.6)	Cycling (9.4, 9.8)	Non-cycling	N/A	2, 5, 7-9
PGM3	Non-cycling	Cycling (4.4)	Cycling (14.7)	Non-cycling	N/A	2-3, 5, 7, 10-11
UAP1	Non-cycling	Cycling (15.9)	Cycling (2, 14.7)	Cycling (22)	N/A	2-5, 7, 10, 12
OGT	Non-cycling	Non-cycling	Cycling (9.2, 12.6, 12.9)	Non-cycling	N/A	2-3, 5, 7, 10, 12-16
OGA	Cycling (22.9)	Cycling (3.8, 8)	Cycling (0, 3.4, 4, 4.5, 5.2, 7.1, 7.5, 7.9, 8.4)	Cycling (8)	N/A	2-4, 7, 9, 11, 13, 17-19

Abbreviations: GFAT/GFPT: Glutamine--fructose-6-phosphate aminotransferase; GNPNAT: Glucosamine-phosphate N-acetyltransferase; PGM3: Phosphoacetylglucosamine mutase; UAP1: UDP-N-Acetyl glucosamine pyrophosphorylase 1; OGT: O-GlcNAc transferase; OGA: O-GlcNAcase; N/A: Undetected. Numbers in parentheses denote peak phase of molecular rhythm.

Supplementary Table 2. Primers for gene expression analysis.

Gene	Forward primer sequences (5'-3')	Reverse primer sequences (5'-3')
gfat1	CCGAGACGTTCTGCAGCTGGAC	GCATGGAGTCGTCTACGAGAGCC
gfat2	GGACGAGCACATTAGGACTGTCCTC	CATCTCCTTGTCCACCAGGGCC
ogt	AGAAGAGCACGTACGACGGGG	TGAGGCTAACGTCTCTCCTGGC
oga	GTACACGGAACTGCGTGAGAAGTAC TCTAG	CGGAGATCAACACGGCAGGGAA GG
cbp20	GTCTGATTCGTGTGGACTGG	CAACAGTTTGCCATAACCCC

Supplementary References

1. Li, Y.H. et al. O-GlcNAcylation of PERIOD regulates its interaction with CLOCK and timing of circadian transcriptional repression. *PLoS Genet.* **15**, e1007953 (2019).
2. Wang, C., et al. Integrated omics in *Drosophila* uncover a circadian kinome. *Nat. Commun.* **11,** 2710 (2020).
3. Rodriguez, J., et al. Nascent-Seq analysis of *Drosophila* cycling gene expression. *Proc. Natl. Acad. Sci. U. S. A.* **110**, E275-84 (2013).
4. Hughes, M. E., et al. Harmonics of circadian gene transcription in mammals. *PLoS Genet.* **5**, e1000442 (2009).
5. Mauvoisin, D., et al. Circadian clock-dependent and -independent rhythmic proteomes implement distinct diurnal functions in mouse liver. *Proc. Natl. Acad. Sci. U. S. A.* **111**, 167–172 (2014).
6. Robles, M. S., Humphrey, S. J. & Mann, M. Phosphorylation is a central mechanism for central control of metabolism and physiology. *Cell Metab.* **25**, 118–127 (2017).
7. Li, X., et al. CirGRDB: a database for the genome-wide deciphering circadian genes and regulators. *Nucleic Acids Res.* **46**, D64–D70 (2018).
8. Du, N. H., Arpat, A. B., De Matos, M. & Gatfield, D. MicroRNAs shape circadian hepatic gene expression on a transcriptome-wide scale. *Elife* **3**, e02510 (2014).
9. Janich, P., Arpat, A. B., Castelo-Szekely, V., Lopes, M. & Gatfield, D. Ribosome profiling reveals the rhythmic liver translatome and circadian clock regulation by upstream open reading frames. *Genome Res.* **25**, 1848–1859 (2015).
10. Kuintzle, R., et al. Circadian deep sequencing reveals stress-response genes that adopt robust rhythmic expression during aging. *Nat Commun* **8,** 14529 (2017).
11. Hughes, M. E., et al. Brain-specific rescue of clock reveals system-driven transcriptional rhythms in peripheral tissue. *PLoS Genet.* **8**, e1002835 (2012).
12. Jouffe, C., et al. The circadian clock coordinates ribosome biogenesis. *PLoS Biol.* **11**, e1001455 (2013).

13. Zhang, R., Lahens, N. F., Balance, H. I., Hughes, M. E., & Hogenesch, J. B. A circadian gene expression atlas in mammals: implications for biology and medicine. *Proc. Natl. Acad. Sci. U. S. A.* **111**, 16219–16224 (2014).
14. Terajima, H., et al. ADARB1 catalyzes circadian A-to-I editing and regulates RNA rhythm. *Nat. Genet.* **49**, 146–151 (2017).
15. Kim, E. Y., et al. A role for O-GlcNAcylation in setting circadian clock speed. *Genes Dev.* **26**, 490–502 (2012).
16. Kaasik, K., et al. Glucose sensor O-GlcNAcylation coordinates with phosphorylation to regulate circadian clock. *Cell Metab.* **17**, 291–302 (2013).
17. Masri, S., et al. Partitioning circadian transcription by SIRT6 leads to segregated control of cellular metabolism. *Cell* **158**, 659–672 (2014).
18. Yang, G., et al. Timing of expression of the core clock gene Bmal1 influences its effects on aging and survival. *Sci. Transl. Med.* **8**, 324ra16 (2016).
19. Atger, F., et al. Circadian and feeding rhythms differentially affect rhythmic mRNA transcription and translation in mouse liver. *Proc. Natl. Acad. Sci. U. S. A.* **112**, E6579–6588 (2015).
