# Supplementary material for: Hexosamine biosynthetic pathway and O-GlcNAc-processing enzymes regulate daily rhythms in protein O-GlcNAcylation
Source: Nat Commun. 2021 Jul 7;12:4173. doi: 10.1038/s41467-021-24301-7 (PMC8263742; doi:10.1038/s41467-021-24301-7)
Supplement: Supplementary file 3 — Description of Additional Supplementary Information [file 41467_2021_24301_MOESM3_ESM.pdf]

Description of Additional Supplementary Files

File Name: Supplementary Data 1

Description: Untargeted metabolomics of *Drosophila* heads and bodies on GC TOF platform.

File Name: Supplementary Data 2

Description: Rhythmicity analysis (RAIN) of untargeted metabolomics.

File Name: Supplementary Data 3

Description: Differential rhythm analysis (DODR) of HBP metabolites in TRF flies.
