# Supplementary material for: Hexosamine biosynthetic pathway and O-GlcNAc-processing enzymes regulate daily rhythms in protein O-GlcNAcylation
Source: Nat Commun. 2021 Jul 7;12:4173. doi: 10.1038/s41467-021-24301-7 (PMC8263742; doi:10.1038/s41467-021-24301-7)
Supplement: Supplementary file 8 — Author’s checklist for requested editorial changes [file 41467_2021_24301_MOESM8_ESM.docx]

Your manuscript has been checked for clarity and against journal policies and formatting style. The issues listed below must be addressed; failure to do so will cause delays in acceptance.

For further information, please see our [formatting instructions](https://www.nature.com/documents/ncomms-formatting-instructions.pdf).

Please highlight all changes in the manuscript text file, either using the track changes feature in Microsoft Word or coloured highlighting in LaTeX.

Please include your response to these requests in the space provided and return this checklist with your final submission.

| **EDITORIAL REQUESTS:** | **AUTHOR RESPONSE:** |
| --- | --- |
| The main manuscript file must be provided in Microsoft Word or LaTeX format. | The main manuscript file is provided in Microsoft Word format. |
| **POLICIES AND CHECKLISTS** | **POLICIES AND CHECKLISTS** |
| An updated editorial policy checklist must be completed and uploaded as a related manuscript file with the revised manuscript. All points on the policy checklist must be addressed; if needed, please revise your manuscript in response to these points. Please note that this form is a dynamic 'smart pdf' and must therefore be downloaded and completed in Adobe Reader, instead of opening it in a web browser. https://www.nature.com/authors/policies/Policy.pdf | Editorial Policy checklist was updated. |
| Please find attached a reporting summary that includes comments on how to revise it in line with our policies and requests the addition of further information in the text. An updated reporting summary must be completed and uploaded as a supplementary information file with the revised manuscript. This checklist is published alongside your manuscript online. Please note that this form is a dynamic 'smart pdf' and must therefore be downloaded and completed in Adobe Reader, instead of opening it in a web browser. https://www.nature.com/authors/policies/ReportingSummary.pdf | Reporting summary was updated as suggested. |
| Please also find below a list of comments requesting additional information in the figure legends, text, and methods section to comply with our reporting policies. | All recommended changes were made. |
| **LANGUAGE AND STYLE (page 6 of our formatting instructions)** | **LANGUAGE AND STYLE (page 6 of our formatting instructions)** |
| Please do not use italics, bold font, underlining or speech marks unless required for technical terms (in both the main text and the display items). | We edited the main text and display items according to the requirements. |
| Please use italics for gene names, and roman font for protein names. This applies to both the main text and display items. |  |
| Please make sure that mathematical terms throughout your manuscript and Supplementary Information (including in figures, figure axes, and legends) conform strictly to the following guidelines. Equations must be supplied in editable format, and not as images. Scalar variables (e.g. x, V, χ) must be typeset in italic, whereas multi-letter variables and functions (e.g. log) must be formatted in roman. Vectors (such as the wavevector k or the magnetic field vector B) must be typeset in bold without italics. | OK |
| **METHODS AND DATA (page 3 of our formatting instructions)** | **METHODS AND DATA (page 3 of our formatting instructions)** |
| Sufficient details of the experiments must be provided in the Methods section such that they could be reproduced without reference to published papers. Use of the term 'as described previously' is not encouraged. | OK |
| A complete list of all primers used, including the names and sequences, must be supplied as a Supplementary Table, which must be cited once in the Methods section. | OK |
| Centrifugation speeds should be described in xg, not rpm. | The units of centrifugation speed were changed to xg. |
| All manuscripts must include a Data Availability statement as a separate section after the Methods section but before the References. For acceptable examples, see: https://www.nature.com/documents/nr-data-availability-statements-data-citations.pdf The Data Availability statement should include:  - Accession codes with hyperlinks for deposited data   - Other unique identifiers (such as DOIs and hyperlinks for any other datasets)   - At a minimum, a statement confirming that all relevant data are available from the authors   - If applicable, a statement regarding data available with restrictions  - If a dataset has a Digital Object Identifier (DOI) as its unique identifier, we strongly encourage including this in the Reference list and citing the dataset in the Data Availability Statement ***Please note that the data must be released publicly by the time you resubmit your final manuscript; we will not be able to accept your manuscript if the data are not publicly available.*** | Hyperlinks were added to the accession codes. The data have been released publicly. |
| Nature Research policies (https://go.nature.com/data-availability-AIP) strongly encourage deposition of research data in public repositories. In some cases this is mandatory, and you may have been previously advised if that was the case. If you need help depositing and curating your research data you should consider: - Contacting Springer Nature’s Research Data Helpdesk (https://go.nature.com/helpdesk-AIP) for advice - Finding a suitable data repository (https://go.nature.com/RD-policies-AIP) for your data Please provide a unique identifier for the data (for example a DOI or a permanent URL) in the data availability statement, if possible. If the repository does not provide identifiers, we encourage authors to supply the search terms that will return the data. For data that have been obtained from publicly available sources, please provide a URL and the specific data product name in the data availability statement. Data with a DOI should be included in the reference list and cited where relevant. Alternatively, include the data in the Supplementary Information. For datasets for which mandatory deposition is not required and the data can only be shared on request, please explain why in your Data Availability Statement and in your response here.  Please refer to our data policies here: http://www.nature.com/authors/policies/availability.html | Date were deposited into public repositories or included as source data in supplemental data file 4. |
| All accession codes must be accompanied with their hyperlinks throughout (for example, "5XRN [http://doi.org/10.2210/pdb5XRN/pdb]", "1483958 [https://doi.org/10.5517/ccdc.csd.cc1lt5m6]", "SRP109982 [https://www.ncbi.nlm.nih.gov/sra/?term=SRP109982]", "GSE101099 [https://www.ncbi.nlm.nih.gov/geo/query/acc.cgi?acc=GSE101099]" or "NQLW00000000 [https://www.ncbi.nlm.nih.gov/assembly/GCA_002312845.1/]"). | Hyperlinks were added to the accession codes. |
| A reference to the source data file should be added in the 'Data Availability' section, using the text “Source data are provided with this paper.” | The sentence “Source data for all figures are provided in Supplementary Data 4” were added to the “Data availability” section. |
| **DISPLAY ITEMS (pages 4 and 5 of our formatting instructions)** | **DISPLAY ITEMS (pages 4 and 5 of our formatting instructions)** |
| The use or adaptation of previously published images is strongly discouraged. If this is unavoidable, please request the necessary rights documentation to re-use such material from the relevant copyright holders and return this to us when you submit your revised manuscript. Please check whether your manuscript or Supplementary Information contain third-party images, such as figures from the literature, stock photos, clip art or commercial satellite and map data. | Noted. |
| In particular, please indicate whether you or a co-author created figures 3a, 7a; Supplementary Fig. 5. | Figure 3a was made using MS PowerPoint by the first author. Figure 7a and Supplementary Fig. 5 were made using BioRender by the first author. |
| Any abbreviations, symbols or colours present in your figures must be defined in the associated legends. | OK |
| All colour scales must be defined and intensity levels must be provided in either the figure or its associated legend. | OK |
| Tables must be black and white, and data must be free from bold/italic formatting unless this has been clearly defined in the footnote. Tables must not include vertical/horizontal/diagonal dividing lines to separate text within the same cell. Text should be split into separate cells and aligned using the appropriate columns and rows (e.g. to put data on a second line). A table must have the same length and width throughout. If not, please ensure that they are made into separate tables and numbered separately. Please format your tables accordingly. | OK |
| Tables must be editable and prepared using the table menu in Word or the table environment in LaTeX. | Tables were prepared using the table menu in Word. |
| **SUPPLEMENTARY INFORMATION (page 5 of our formatting instructions)** | **SUPPLEMENTARY INFORMATION (page 5 of our formatting instructions)** |
| We do not edit Supplementary Information files; they will be uploaded with the published article as they are submitted with the final version of your manuscript. Any tracked changes should be removed from the file and the file should be provided as a PDF file. Supplementary Figures do not need to be provided separately. | OK |
| Supplementary References should appear at the end of the Supplementary Information file, and must be self-contained and numbered from 1. References mentioned in both the main text and the Supplementary Information should be part of both reference lists so that the Supplementary Information does not refer to the reference list in the main paper and vice versa. | OK |
| Please supply legends for each Supplementary Movie/Audio/Data file in your response here (not in the Supplementary Information file). Please label each files as Supplementary Movie/Audio/Data 1, etc. | We have four supplementary data files:  Supplementary Data 1. Untargeted metabolomics of *Drosophila* heads and bodies on GC TOF platform.  Supplementary Data 2. Rhythmicity analysis (RAIN) of untargeted metabolomics.  Supplementary Data 3. Differential rhythm analysis (DODR) of HBP metabolites in TRF flies.  Supplementary Data 4. Source Data |
| Please ensure that a Source Data file is included with your resubmission. Within the Source Data file, the relevant raw data from each figure or table (in the main manuscript and in the Supplementary Information) should be represented by a single sheet in an Excel document, or a single .txt file or other file type in a zipped folder. Uncropped blots and gel images should be pasted in and labelled with the relevant panel and identifying information such as the antibody used. An example of the Source Data file is available demonstrating the correct format: https://www.nature.com/documents/ncomms-example-source-data.xlsx The file should be labelled 'Source Data', with the title and a brief description included in your response here, and should be mentioned in all relevant figure legends using the template text below: "Source data are provided as a Source Data file." | A Source Data file with the raw data for each figure and uncropped western blots is provided as Supplemental Data File 4. |
| **PUBLICATION** | **PUBLICATION** |
| In addition, please supply a two sentence summary of your work to accompany the paper on our homepage. The summary should be accessible to a broad audience, contain no more than 250-300 characters including spaces, and should include two sentences, the first of which describes the background to the work, and the second of which summarises the major conclusions. | Misalignment between human lifestyles and natural day-night cycles, such as mistimed eating, can negatively impact healthspan. This study shows that mistimed feeding alters protein O-GlcNAcylation, a nutrient sensitive post-translational modification, and contributes to disrupted circadian rhythms. |
| As part of our efforts to communicate our content to a wider audience, we endeavour to highlight papers published in Nature Communications on the journal’s Twitter account (https://twitter.com/NatureComms). If you would like us to mention authors, institutions or lab groups in these tweets, please provide the relevant twitter handles. | Please mention these authors on twitter: Xianhui Liu (@NitrolLiu) and Joanna Chiu (@joanna_c_chiu). Please also include @UCDavisResearch |

|  | **EDITORIAL REQUESTS:** | **AUTHOR RESPONSE:** |
| --- | --- | --- |
| **1.** | **Data presentation:** Please ensure that data presented in a plot, chart or other visual representation format shows data distribution clearly (e.g. dot plots, box-and-whisker plots). When using bar charts, please overlay the corresponding data points (as dot plots) whenever possible and always for n ≤ 10. (Please see the following editorial for the rationale behind this request and an example <https://www.nature.com/articles/s41551-017-0079>). | |
| **2.** | **Statistics**: Wherever statistics have been derived (e.g. error bars, box plots, statistical significance) the legend needs to provide and define the n number (i.e. the sample size used to derive statistics) as a precise value (not a range), using the wording “n=X biologically independent samples/animals/cells/independent experiments/n= X cells examined over Y independent experiments” etc. as applicable. | |
| **3.** | Please note that statistics such as error bars significance and p values cannot be derived from n<3 and must be removed in all such cases. | |
|  | We strongly discourage deriving statistics from technical replicates, unless there is a clear scientific justification for why providing this information is important. Conflating technical and biological variability, e.g., by pooling technically replicates samples across independent experiments is strongly discouraged. (For examples of expected description of statistics in figure legends, please see the following <https://www.nature.com/articles/s41467-019-11636-5> or <https://www.nature.com/articles/s41467-019-11510-4>). | |
|  | All error bars need to be defined in the legends (e.g. SD, SEM) together with a measure of centre (e.g. mean, median). For example, the legends should state something along the lines of “Data are presented as mean values +/- SEM” as appropriate.  All box plots need to be defined in the legends in terms of minima, maxima, centre, bounds of box and whiskers and percentile. | |
|  | **Legends requiring revision:**   1. Please note that the error bars need to be defined in the legends of figures 3a, b. | The error bars of figures 3a, b are now defined as SEM in the legends. |
| **4.** | The figure legends must indicate the statistical test used. Where appropriate, please indicate in the figure legends whether the statistical tests were one-sided or two-sided and whether adjustments were made for multiple comparisons.  For null hypothesis testing, please indicate the test statistic (e.g. F, t, r) with confidence intervals, effect sizes, degrees of freedom and P values noted.  Please provide the test results (e.g. P values) as exact values whenever possible and with confidence intervals noted. | |
|  | **Legends requiring revision:**   1. Please indicate the statistical test used for data analysis and where appropriate, please specify whether it was one-sided or two-sided and whether adjustments were made for multiple comparisons, in the legends of figures 1b; 2a, b, e; 3e, f; 4a; 5b, c, e-j; 6b, c; supplementary figures 4g, h; and in the legends of supplementary data 2; 3. 2. Please note that the exact p value should be provided, when possible, in the legends of supplementary figures 4b-d. 3. Please note that for the figures 1b; 2a, b; 5b, c, e-j; 6b, c; supplementary figures 4g, h, p-values and statistical tests are indicated in the legends. However, comparison for the same, has not been represented in the figures. Please rectify this in the figures or legends as applicable. | 1. We indicated the statistical tests in the figure legends and legends for supplementary data. RAIN (Rhythmicity Analysis Incorporating Nonparametric methods), DODR (Detection of Differential Rhythmicity) or CircaCompare statistical tests are for testing 24-hour rhythmicity. They are not one-sided or two-sided tests, and no adjustments were made for these tests. RAIN is a nonparametric statistical test to describe the rhythmicity of curves; RAIN utilizes umbrella method and Mann–Whitney U test to evaluate the rise and down of rhythmic data. DODR and CircaCompare are statistical tests to compare the rhythmicity of data. DODR uses Mann–Whitney U test to estimate the phase or amplitude difference between curves. CircaCompare uses cosinusoidal curve fitting method to obtain parameters and therefore compares the amplitude, phase, and mesor of rhythms for two conditions. 2. For supplementary figures 4b-d, the statistical tests (two-way ANOVA with post-hoc Tukey's HSD tests) were performed in GraphPad Prism 8.0. The exact p values were not provided by the software because p values were too small (p < 0.0001). 3. For figures 1b; 2a; 5b, c, e-j; 6b, c; supplementary figures 4g, h, the p values indicate the rhythmicity of data sets (RAIN) or the comparison of rhythmicity between conditions (DODR or Circacompare). We feel it is confusing and not appropriate to use asterisks to indicate the significance of data in those cases, as we use asterisks to indicate the significance level at individual time point in other figure panels, such as supplementary figure 4b-d. Finally, as figure 2b illustrates the percentages of cycling v.s. non-cycling compounds determined based on RAIN test, the exact p value or asterisk symbols are not suitable for this panel. |
| **5.** | **Reproducibility:** Please state in the legends how many times each experiment was repeated independently with similar results. This is needed for all experiments, but is particularly important wherever results from representative experiments (such as micrographs) are shown. If space in the legends is limiting, this information can be included in a section titled “Statistics and Reproducibility” in the methods section. | |
|  | **Legends requiring revision:**  Please note that this information is missing in the legends of figures 3d; 5a, d; 6a; supplementary figures 1a, c-f; 4f. | The information was added to the legends of figures 3d; 5a, d; 6a; supplementary figures 1a, c-f; 4f. |
| **6.** | **Data availability:** This journal strongly supports public availability of data and custom code associated with the paper in a persistent repository where they can be freely and enduringly accessed or as a supplementary data file when no appropriate repository is available. If data and code can only be shared on request, please explain why in your data Availability Statement, and also in the correspondence with your editor. For more information, please refer to <https://www.nature.com/nature-research/editorial-policies/reporting-standards#availability-of-data> | |
|  | Please ensure that datasets deposited in public repositories are now publicly accessible, and that accession codes or DOI are provided in the "Data Availability" section. As long as these datasets are not public, we cannot proceed with the acceptance of your paper. For data that have been obtained from publicly available sources, please provide a URL and the specific data product name in the data availability statement. Data with a DOI should be further cited in the methods reference section. | The deposited metabolomics data have been released publicly and accession codes were provided in the "Data Availability" section. For data from public sources, URL and the specific data product name were provided in the "Data Availability" section. |
| **7.** | **Gels and Blots:** Quantitative comparisons between samples on different gels/blots are discouraged; if this is unavoidable, the figure legend must state that the samples derive from the same experiment and that gels/blots were processed in parallel.  Vertically sliced images that juxtapose lanes that were non-adjacent in the gel must have a clear separation or a black line delineating the boundary between the gels. Loading controls (e.g. GAPDH, actin) must be run on the same blot.  Sample processing controls run on different gels must be identified as such in the figure legends, and distinctly from loading controls.  All blots and gels must be accompanied by the locations of molecular weight/size markers. Blots should be cropped such that at least one marker position is present.  Please also supply uncropped and unprocessed scans of the most important blots in the Source Data file or as a supplementary figure in the Supplementary Information. This should be cited once in the Methods section. For an example of presentation of full scan blots, see the Source Data file of <https://www.nature.com/articles/s41467-020-16984-1#Sec35> and for more information, please refer to <https://www.nature.com/nature-research/editorial-policies/image-integrity> | |
|  | **Panels requiring revision:**   1. Please note that molecular weight markers are missing for figures 1a; 3d; 5a, d; 6a; supplementary figures 1a, c-f; 4f. | Molecular weight markers were added to figures 1a; 3d; 5a, d; 6a; supplementary figures 1a, c-f; 4f. |
